# *Bartonella* and *Rickettsia* in arthropods from the Lao PDR and from Borneo, Malaysia^☆^

**DOI:** 10.1016/j.cimid.2011.10.003

**Published:** 2012-01

**Authors:** Tahar Kernif, Cristina Socolovschi, Konstans Wells, Maklarin B. Lakim, Saythong Inthalad, Günther Slesak, Najma Boudebouch, Jean-Claude Beaucournu, Paul N. Newton, Didier Raoult, Philippe Parola

**Affiliations:** a Unité de Recherche sur les Maladies Infectieuses et Tropicales Emergentes URMITE, IFR48, CNRS 6236, IRD 198, Faculté de Médecine, Université de la Méditerranée, Marseille, France; b Biodiversity and Climate Research Centre (Bik-F), Senckenberganlage 25, D-60325 Frankfurt (Main), Germany; c Institute of Experimental Ecology, Albert-Einstein-Allee 11, University of Ulm, Germany; d Sabah Parks, Kota Kinabalu, Sabah, Malaysia; e Luang Namtha Provincial Hospital, Luang Namtha, Lao Democratic People's Republic; f Service Fraternel d’Entraide (SFE) Medical Project, Luang Namtha Project, SFE, P.O. Box 5211, Vientiane, Lao Democratic People's Republic; g Tropenklinik Paul-Lechler-Krankenhaus, Paul-Lechler-Str. 24, 72076 Tübingen, Germany; h Laboratoire des Maladies Vectorielles, Institut Pasteur du Maroc, Maroc; i Laboratoire de Parasitologie et Zoologie Appliquée, Faculté de Médecine, Rennes, France; j Wellcome Trust-Mahosot Hospital-Oxford Tropical Medicine Research Collaboration, Microbiology Laboratory, Mahosot Hospital, Vientiane, Lao Democratic People's Republic; k Centre for Tropical Medicine, Nuffield Department of Clinical Medicine, University of Oxford, Churchill Hospital, Oxford, OX3 7LJ, UK

**Keywords:** Siphonaptera, Anoplura, Acarina, *Rickettsia*, *Bartonella*, Laos, Borneo Island, Zoonotic disease transmission, Spotted fever

## Abstract

Rickettsioses and bartonelloses are arthropod-borne diseases of mammals with widespread geographical distributions. Yet their occurrence in specific regions, their association with different vectors and hosts and the infection rate of arthropod-vectors with these agents remain poorly studied in South-east Asia. We conducted entomological field surveys in the Lao PDR (Laos) and Borneo, Malaysia by surveying fleas, ticks, and lice from domestic dogs and collected additional samples from domestic cows and pigs in Laos. *Rickettsia felis* was detected by real-time PCR with similar overall flea infection rate in Laos (76.6%, 69/90) and Borneo (74.4%, 268/360). Both of the encountered flea vectors *Ctenocephalides orientis* and *Ctenocephalides felis felis* were infected with *R. felis*. The degrees of similarity of partial *gltA* and *ompA* genes with recognized species indicate the rickettsia detected in two *Boophilus* spp. ticks collected from a cow in Laos may be a new species. Isolation and further characterization will be necessary to specify it as a new species. *Bartonella clarridgeiae* was detected in 3/90 (3.3%) and 2/360 (0.6%) of examined fleas from Laos and Borneo, respectively. Two fleas collected in Laos and one flea collected in Borneo were co-infected with both *R. felis* and *B. clarridgeiae.* Further investigations are needed in order to isolate these agents and to determine their epidemiology and aetiological role in unknown fever in patients from these areas.

Since the beginning of the 20th century, ticks (Acarina), lice (Anoplura and Mallophaga) and fleas (Siphonaptera) have been implicated as vectors, reservoirs, and/or amplifiers of agents of human zoonoses, including rickettsioses and bartonelloses [14]. These diseases have been poorly investigated in South-east Asia, including the Lao PDR (Laos) [2,16] and Malaysian Borneo [10]. Among hospitalized patients in Vientiane, Laos, acute rickettsial infection was identified as the cause of fever in115 (27%) of 427 adults [16] and cause of jaundice or hepatitis in 29 (7.3%) from 392 patients admitted in Mahosot Hospital [27]. The organisms identified by serological analysis were *Orientia tsutsugamushi*, *Rickettsia typhi*, and spotted fever group *Rickettsia* (SFGR) (*R. helvetica*, *R. felis*, *R. conorii* subsp. *indica*, and *Rickettsia* “AT1”) [16,27]. In addition, *Bartonella clarridgeiae* and *Rickettsia felis* were detected in fleas collected in Phu Khao Khoay, near Vientiane [30]. A clinical case of murine typhus (*R. typhi*) was reported in a traveller from Brunei [9] and *R. felis* and *R. typhi* have been detected from flea species associated with small mammals in these area [3]. A recent study on flea-host associations, for example, compiled no more than 15 fleas species described to date to occur on small terrestrial mammals on Borneo Island, highlighting the lack of our understanding of the species diversity of possible vector and their role in transmitting diseases [32]. As part of our recent efforts to better understand the diversity, occurrence and distribution of ectoparasites of medical importance in South-East Asia, we collected ectoparasites from domestic dogs and some additional samples from domestic cows and pigs in Laos. We investigated the collected arthropods for *Rickettsia* and *Bartonella* species in order to examine which species of these disease-causing groups are present in northern Laos and Sabah, East Malaysia, on Borneo Island.

## 1 Materials and methods

### 1.1 Study sites and sampling

**Fig. 1:**
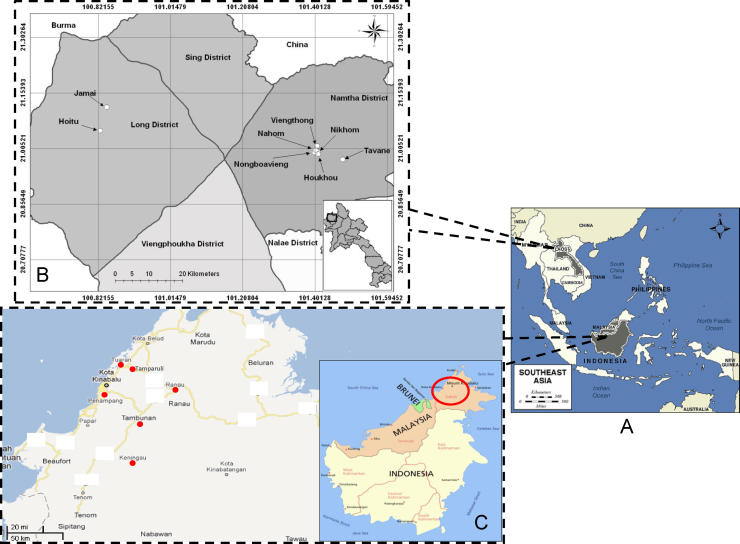
(A) Location of Laos and Borneo Island in South-East Asia. (B) The sample sites where fleas, ticks and lice have been collected in Luang Namtha Province, Laos. (C) The sample sites where fleas have been collected in the state of Sabah from Malaysia, Borneo Island.

In Luang Namtha Province (20°55′N, 101°07′E), northwest Laos, ectoparasites were sampled in eight villages, including Tavane, Nikhom, Viengthong, Jamai, Hoitu, Houkhou, Nongbauvieng and Nahom (Fig. 1A and B).

On Borneo (6°02′N, 116°7′E), in the state of Sabah, Malaysia, arthopods were collected near the capital town Kota Kinabalu and in the districts of Keningau, Penampang, Tambunan, Tamparuli, Tuaran, and Ranau which are near the Crocker Range National Park and Kinabalu National Park (Fig. 1C). We particularly focused on these sites as this study was part of our initial efforts to investigate possible interactions of wildlife and domestic animals and the potential of zoonotic diseases transmission near protected forests.

### 1.2 Collections and morphological identification of ectoparasites

In Luang Namtha Province, northwest Laos, fleas, ticks and lice were collected from domestic dogs, pigs and cows. In Borneo Island, fleas were only collected from domestic dogs. People encountered while travelling through the study area were asked for access to their dogs. We sampled only one dog per household of those consenting; usually, the sampled dog was chosen by the house owner based on availability and tameness. We brushed the dorsal fur of dogs from the neck to the tail for ten minutes with a flea comb (Trixie, Tarp, Germany, art. no. 23762). All ectoparasites were transferred with forceps to a tube containing 70% ethanol for later counting and identification and transported to France. All ectoparasites were morphologically identified to the species level by using morphological criteria within standard taxonomic keys [4,28].

### 1.3 Molecular analysis

DNAs of ectoparasites were extracted using the BioRobot MDx Workstation (Qiagen, Courtaboeuf, France) with a customized extraction protocol following the manufacturer's instructions. For DNA extraction, a negative control (one non-infected tick from laboratory colony) was used for each 15 samples. DNA was stored at 4 °C until use for further analysis.

Real-time quantitative (q)PCR was performed according to the manufacturer's protocol using a 7900HT Fast Real-Time PCR system (Applied Biosystems, Foster City, CA). Positive samples were considered when cycle thresholds were Ct ≤ 35. All samples were screened using *SFGR*-specific qPCR targeting *glt*A gene [25] and *Bartonella* genus-specific real-time PCR with a 21-bp probe targeting the intergenic spacer (ITS) [30]. Positive fleas for *Rickettsia* DNA were tested subsequently with primers and probe targeting a chromosomal gene specific of *R. felis bioB*, as described previously [30]. Positive ticks for *Rickettsia* DNA were tested by regular PCR targeting *gltA* and *Omp*A genes [23]. *Bartonella* positive samples were tested by regular PCR using primers amplifying ITS at fragment [15]. The *Bartonella* and *Rickettsia* DNA sequencing reagents were obtained with BigDye Terminator Cycle Sequencing Ready Reaction Kit (ABI PRISM, PE Applied Biosystems, Foster City, CA). The resulting sequences were edited and assembled using Chromas Pro 1.34 (Technelysium Pty. Ltd., Tewantin, Australia). The sequences were then analyzed by Basic Local Alignment Search Tool (BLAST) sequencing and further compared with other sequences available in the GenBank. *R. montanensis*, *R. felis*, and *B. elizabethae* DNA were included as positive control. Two negative controls were used for each test: sterile water and DNA extracted from non-infected ticks taken from a colony at the Unité des Rickettsies, Marseille.

We used Fisher's Exact Test for categorical data as implemented in the software package R [17].

## 2 Results

### 2.1 Collection and morphological identification of the ectoparasites

In Laos, a total of 90 fleas were collected from ten dogs in five villages, including Tavane, Nikhom, Viengthong, Jamai and Hoitu. These fleas were morphologically identified as *Xenopsylla cheopis* (*n* = 2), *Pulex irritans* (*n* = 3), *Ctenocephalides felis felis,* the cat flea (*n* = 19), and *Ctenocephalides orientis*, the Asian cat flea (*n* = 66).

Ninety-three ticks were collected, comprising 39 (41.9%) adults and 54 (58.1%) nymphs. Of the 39 adult ticks, 36 (92.3%) were identified as *Rhipicephalus sanguineus* collected from ten dogs, 2 (5.1%) *Boophilus* spp. collected from a one domestic cow and 1 (2.6%) *Dermacentor* spp. collected from one domestic pig. All nymphs (*n* = 54) were identified as *Rhipicephalus* spp. and were collected from seven dogs (Table 1). These ticks were collected in four villages, including Viengthong, Houkhou, Nongbauvieng and Nahom. Forty lice were collected on 12 pigs in Tavan region. All lice were identified as *Haematopinus suis*, based on their morphological characters.

In Sabah, 1968 fleas were collected from 212 dogs. They were identified as *Ctenocephalides orientis* (75%, 106 out of 142 identified specimens), and *Ctenocephalides felis felis* (25%, 36 out of 142 identified specimens). The mean (±SD) number of fleas collected per individual dog was 9.3 ± 10.4 (max: 67 fleas on one dog, no fleas were found on 17 individuals). Not all fleas collected from Borneo Island were tested, we limited our molecular analysis to a maximum of five fleas per sampled dog and a total of 360 samples.

### 2.2 Rickettsia detection

Rickettsial DNA was detected in 69 of 90 (76.6%) fleas collected from Laos. All fleas positive for rickettsial DNA were positive by *R. felis*-specific qPCR. The frequency of *R. felis* was significantly higher in *C. f. orientis* (59/66; 89.4%) than in *C*. *f. felis* (10/19; 52.6%) (Fisher ‘s test odds ratio 0.14, *p* = 0.001). No *R. felis* DNA was detected in *X. cheopis* and *P. irritans* fleas. *R. felis* DNA was detected in fleas from five out of eight villages for our study (Table 1). Flea infection rates in these five villages ranged from 55% to 100% with significantly lower infection rate in Viengthong than other villages (Fisher's test *p* = 0.03; Table 2). Rickettsial DNA was detected in two *Boophilus* spp. ticks (2.1%; 2/93) collected on a cow in Houkhou. Sequence analysis of the *omp*A gene showed 99.83% (613/614) similarity with *Rickettsia* sp. FUJ98 (GenBank accession no. AF169629) and 95.29% similarity (588/617) with *Rickettsia heilongjiangensis* 054 (GenBank accession no. CP002912). Sequence analysis of *gltA* gene showed 100% (662/662) similarity with *Rickettsia* sp. LON-13 (GenBank accession no. AB516964) and 99.54% similarity (659/662) with *R. heilongjiangensis* (GenBank accession no. AB473994). No rickettsial DNA was detected in *Haematopinus suis* lice.

In Borneo Island, Rickettsial DNA was detected in 268 of 360 samples (74.4%) individually tested from 90 dogs. All fleas positive for rickettsial DNA were positive by *R. felis*-specific qPCR. Notably, *R. felis* appeared to be ubiquitously present throughout the study area in Borneo as we were not able to find rickettsial DNA in only 3 of the 90 dogs for which the fleas collected on them were screened. The overall infection rates of fleas with rickettsial DNA in Laos (76.6%) and Borneo (74.4%) were similar (Fisher's exact test, odds ratio = 1.13, *p* = 0.79).

### 2.3 *Bartonella* detection

In ectoparasites from Laos, *Bartonella* DNA was detected in 3 out of 90 (3.3%) fleas, including only *C. f. orientis* fleas collected from dogs in Tavan and Hoitu (Table 1). Sequences obtained after PCR amplification and sequencing of partial ITS showed 100% identity with *B. clarridgeiae* (GenBank accession no. EU589237). Moreover, two fleas collected in Hoitu were co-infected by *B. clarridgeiae* and *R. felis.* No *Bartonella* DNA was detected in any ticks or lice.

*Bartonella* DNA was detected in 2 (2/360, 0.6%) fleas collected from dogs from Borneo. Sequences obtained after PCR amplification and sequencing of partial ITS showed 99.29% (702/707) identity with *B. clarridgeiae* (GenBank accession no. FN645454). Moreover, one flea was co-infected by *B. clarridgeiae* and *R. felis.*

## 3 Discussion

These findings provide the first evidence of *Rickettisa felis* in Malaysian Borneo and confirm the presence of this agent among ectoparasites in several provinces in Laos with high overall rates of infection of similar magnitude in Laos and Malaysian Borneo. Recently, *R. felis* has been detected in East Kalimantan (Indonesian Borneo) in pools of *Xenopsylla cheopis* collected from the shrew, *Suncus murinus* and the rat *Rattus norvegicus* [3]. These findings suggest that flea-borne spotted fever is likely to be a human disease in these areas and urges that *R. felis* should be included in the differential diagnosis of fever. In 1986, a serological survey in Sabah, East Malaysia showed that 16.5% of 412 human forest dwellers presented SFGR antibody [29]. Differences in flea infection rates among villages in Laos suggests that geographical heterogeneity in human disease risk is likely to occur, although our relatively small sample size for Laos did not allow a comprehensive spatial analysis and the prediction of flea abundance and infections rates under different environmental conditions as would be desirable. Moreover, flea abundance on dogs in Borneo differed with environmental conditions, mostly affected by housing conditions (K. Wells, unpublished data). We found some weak spatial structure in the prevalence of *R. felis* in fleas collected in Borneo (exploratory analysis with a variogram, results not shown), and we emphasize the need for further interdisciplinary studies that investigate pathogen spread under different environmental conditions likely to affect host and vector species.

*R. feli*s has been associated with various species of fleas and human cases have been reported from North and South America, Europe, Asia, and North Africa [13]. Recently, a high prevalence of *R. felis* in dogs in Southeast Queensland, had suggested that dogs may act as an important reservoir host for *R. felis* and as a potential source of human rickettsial infection [7]. The animal hosts from which the infected ectoparasites were recovered represent a large range of different mammalian host species [18]. SE Asia rainforest are hotspots of mammalian species diversity and due to rapid economic growth, further factors that may be relevant to disease emergence such as housing conditions for companion animals, shifts in the abundance and community composition of wildlife species, land cover and exposure of domestic animals to wildlife and vice versa are rapidly changing [24,26,31]. Considerable research efforts are therefore needed to examine the epidemiology of *R. felis* and its relationship to particular regional environmental conditions. In 2007, the first evidence of *R. felis* was reported on fleas collected in dogs from Phu Khao Khoay area (∼100 km NE of Vientiane) from Laos [30]. In addition, *R. felis* infection was diagnosed in patient with unknown fever in Vientiane [16]. Recently, two studies conducted in Senegal and Kenya, have highlighted the importance of *R. felis* infection in patients with unexplained fever in sub-Saharan Africa [13,19,25].

In our study, the degrees of similarity of partial *gltA* and *ompA* genes with recognized species indicate the *Rickettsia* detected in ticks from Laos may be a new species. This *Boophilus* spp. tick *Rickettsia* is a species of the SFGR because its sequence exhibits > 92.7% homology with the 20 known *Rickettsia* species from this group and possess the *omp*A gene [6]. The closest validated *Rickettsia* species to our presumably new species is *R. heilongjiangensis,* the agent of far-eastern tick-borne disease, which occurs in eastern Russia and northern China [14]. However, only fragments of *gltA* and *ompA* genes have been sequenced in our study. To better describe this *Rickettsia* in detail, it will need to be isolated and the five genes fully specific to *Rickettsia* (rrs, *glt*A, *omp*A, *omp*B, and geneD) characterized [6]. Finally, other *Rickettsia* species transmitted by tick-vector circulate in Borneo, as tick typhus was detected by serology among people in Sarawak, East Malaysia [21].

In addition, we report the first detection of *Bartonella clarridgeiae* in fleas collected in Borneo and confirm its presence in Laos. *B. clarridgeiae* has been suggested as a minor causative agent of cat scratch disease (CSD) in humans [8]. The most frequent agent of CSD is *B. henselae* with its principal reservoir being domestic cats [1]. *B. clarridgeiae* has been detected on fleas from Europe, Asia, North America [22], Africa, New Zealand and very recently from New Caledonia [11]. The prevalence of *B. clarridgeiae* in cat fleas in Europe may be as great as 17% [20]. Nearby Laos, *B. clarridgeiae* has been identified on the Thai-Myanmar (Burma) border and in China [15,22] and it has been reported on fleas collected in dogs in Phu Khao Khoay in Laos [30]. A high prevalence of *Bartonella* spp. (25.5%), including *B. elizabethae*, *B. tribocorum*, *B. phoceensis* and two new *Bartonella* species, was reported in rodents collected in four Lao provinces [2]. To the best of our knowledge, no patients with *Bartonella* infection have been reported from Laos or Borneo. The infection rates in our study are relatively low, but given the ubiquitous presence of the vectors, we believe that it is possible that some fevers in these areas may be caused by *Bartonella* spp. A high *Bartonella* seroprevalence in rural Thailand has been reported recently in febrile (10%) and non-febrile patients (19%) [5]. Fatal human *Bartonella* endocarditis confirmed by serology, by qPCR, cell culture, and by immunohistochemical staining has recently been reported in Thailand [12].

In conclusion, our data show a high proportion of fleas being infected with *R. felis* and further report the presence of *B. clarridgeiae* in fleas from both Laos and Borneo. Infections due to these pathogens are likely to be underestimated and misdiagnosed, and should be considered in the differential diagnosis of unknown fever. Beside the importance of considering the pathogens in medical diagnosis, we highlight that more interdisciplinary research is necessary to perform epidemiological studies in the future that investigate further the species diversity of pathogens, their prevalence and also the role of components involved in disease emergence and transmission such as vectors, vertebrate hosts and the underlying environment.

## Conflict of interests

The authors declared that they have no competing interests.

## Figures and Tables

**Table 1:** The frequency of *Rickettsia* and *Bartonella* species in various flea and tick species sampled in Lao PDR and Sabah (Malaysia), Borneo Island.

	Animal species (number of individuals sampled)	Number (percentage) of positive pathogen detection/number of vertebrate host individuals with positives		
		*Rickettsia* spp.	*Rickettsia felis*	*Bartonella clarridgeiae*
Luang Namtha Province, Laos				
Fleas species	Host species			
*Ctenocephalides orientis* (*n* = 66)	Dog (*n* = 7)	59 (89.4%)/*n* = 7	59 (89.4%)/*n* = 7	3 (4.5%)/*n* = 2
*Ctenocephalides felis felis* (*n* = 19)	Dog (*n* = 3)	10 (52.6%)/*n* = 2	10 (52.6%)/*n* = 2	–/*n* = 0
*Pulex irritans* (*n* = 3)	Dog (*n* = 2)	–/*n* = 0	–/*n* = 0	–/*n* = 0
*Xenopsylla cheopis* (*n* = 2)	Dog (*n* = 1)	–/*n* = 0	–/*n* = 0	-–/*n* = 0

Total	Dog (*n* = 10)	69 (76.6%)/*n* = 7	69 (76.6%)/*n* = 7	3 (3.3%)^b^/*n* = 2

Ticks species	Host species			
*Rhipicephalus sanguineus* (*n* = 39)	Dog (*n* = 10)	–/*n* = 0	–/*n* = 0	–/*n* = 0
*Rhipicephalus* spp. (*n* = 54)	Dog (*n* = 7)	–/*n* = 0	–/*n* = 0	–/*n* = 0
*Dermacentor* spp. (*n* = 1)	Pig (*n* = 1)	–/*n* = 0	–/*n* = 0	–/*n* = 0
*Boophilus* spp. (*n* = 2)	Cow (*n* = 1)	2(100%)^a^/*n* = 1	–/*n* = 0	–/*n* = 0

Total	Dog (*n* = 10); Pig (*n* = 1), Cow (*n* = 1)	2 (2.1%)/*n* = 1	–	–

Lice species	Host species			
*Haematopinus suis* (*n* = 40)	Pig (*n* = 12)	–/*n* = 0	–/*n* = 0	–/*n* = 0
Sabah (Malaysia), Borneo Island				
Fleas species				
*Ctenocephalides orientis* and *Ctenocephalides felis felis* (*n* = 360)	Dogs (*n* = 90)	–/*n* = 0	268 (74.4%)/*n* = 87	2 (0.6%)^c^/*n* = 2

a *Rickettsia* sequences: sequence analysis of *omp*A gene [99.83% similarity with *Rickettsia* sp. FUJ98 (GenBank accession no. AF169629) and 95.29% similarity with *Rickettsia heilongjiangensis* 054 (GB no. CP002912)]; *gltA* gene [100% similarity with *Rickettsia* sp. LON-13 (GB no. AB516964) and 99.54% similarity with *R. heilongjiangensis* (GB no. AB473994)].

b *Bartonella* sequences: sequence analysis of the intergenic spacer ITS showed 100% identity with *B. clarridgeiae* (GB no. EU589237).

c *Bartonella* sequences: sequence analysis of the intergenic spacer ITS showed 99.27% identity with *B. clarridgeiae* (GB no. FN645454).

**Table 2:** Distribution of *Rickettsia* and *Bartonella* spp. in vectors (fleas, ticks) sampled at the different sites of Luang Namtha Province from Laos and in different districts of Sabah (Malaysia), Borneo Island.

Regions	Positive for *Rickettsia* in qPCR/number collected/%		Probe *R. felis bioB* gene (fleas)	Positive for *Bartonella* (qPCR)/number collected (%)	
	Fleas	Ticks		Fleas	Ticks
Luang Namtha Province, Laos					
Tavane	36/42 (85.7%)	–	36	1/42 (4.7%)	–
Nikhom	15/23 (65.2%)	–	15	0/23	–
Viengthong	11/20 (55%)	0/22	11	0/20	0/22
Jamai	5/5 (100%)	–	5	0/5	–
Hoitu	2/2 (100%)	–	2	2/2 (100%)	–
Houkhou	–	2/8 (25%)	–	–	0/8
Nongbauvieng	–	0/39	–	–	0/39
Nahom	–	0/24	–	–	0/24
Total (%)	69/90 (76.6%)	2/93 (2.1%)	69	3/90 (3.3%)	0/93
Sabah, (Malaysia), Borneo Island					
Keningau	81/105 (77%)	–	81	–	–
Kota Kinabalu	15/17 (88%)	–	15	–	–
Penampang	15/20 /75%)	–	15	–	–
Tambunan	100/158 (63%)	–	100	1/158 (0.6%)	–
Tamparuli	31/34 (91%)	–	31	1/34 (3%)	–
Tuaran	26/26 (100%)	–	26	–	–
Ranau	0/0	–	0	–	–
Total (%)	268/360 (74.5%)	–	268	2/360 (0.6%)	–
